# 1068. Prior SARS-CoV-2 infection and risk of subsequent COVID-19-related hospitalization: a test negative design

**DOI:** 10.1093/ofid/ofac492.909

**Published:** 2022-12-15

**Authors:** Khalel De Castro, Ashley Tippett, Laila Hussaini, Luis W Salazar, Olivia D Reese, Meg Taylor, Caroline R Ciric, Chris Choi, Grace Taylor, Laura A Puzniak, Robin Hubler, Srinivas Valluri, Benjamin Lopman, Satoshi Kamidani, Christina A Rostad, John M McLaughlin, Evan J Anderson

**Affiliations:** Emory University, Atlanta, Georgia; Emory University, Atlanta, Georgia; Emory Univeristy, Atlanta, Georgia; Emory University, Atlanta, Georgia; Emory University, Atlanta, Georgia; Emory University, Atlanta, Georgia; Emory University, Atlanta, Georgia; Emory University, Atlanta, Georgia; Emory University, Atlanta, Georgia; Pfizer Inc., Collegeville, Pennsylvania; Pfizer Inc., Collegeville, Pennsylvania; Pfizer Inc, New York, New York; Rollins School of Public Health | Emory University, Atlanta, Georgia; Emory University School of Medicine and Children's Healthcare of Atlanta, Atlanta, Georgia; Emory University School of Medicine and Children's Healthcare of Atlanta, Atlanta, Georgia; Pfizer, Collegeville, Pennsylvania; Emory University School of Medicine, Atlanta, Georgia

## Abstract

**Background:**

Studies show that past SARS-CoV-2 infection provides a protective immune response against subsequent COVID-19, but the degree of protection from prior infection has not been determined.

History of previous SARS-COV-2 Infection and Current SARS-COV-2 Infection Status at Admission.

**Figure:**
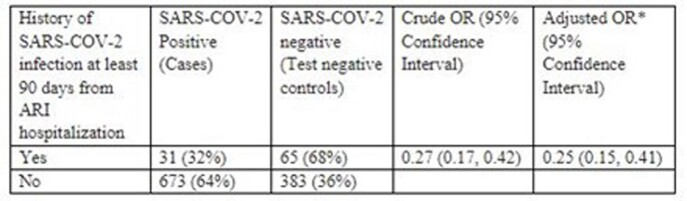

*Adjusted for chronic respiratory disease and prior COVID-19 vaccination

**Methods:**

From May 2021 through Feb 2022, adults (≥ 18 years of age) hospitalized at Emory University Hospital and Emory University Hospital Midtown with acute respiratory infection (ARI) symptoms, who were PCR tested for SARS-CoV-2 were enrolled. A prior history of SARS-CoV-2 infection was obtained from patient interview and medical record review. Previous infection was defined as a self-reported prior SARS-CoV-2 infection or previous evidence of a positive SARS-CoV-2 PCR test ≥ 90 days before ARI hospital admission. We performed a test negative design to evaluate the protection provided by prior SARS-CoV-2 infection against subsequent COVID-19-related hospitalization. Effectiveness was determined using logistic regression analysis adjusted for patient sociodemographic and clinical characteristics and COVID-19 vaccination status.

**Results:**

Of 1152 adults hospitalized for ARI, 704/1152 (61%) were SARS-CoV-2 positive. 96/1152 (8%) had a prior SARS-CoV-2 infection before hospital admission. Patients with a previous history of SARS-CoV-2 infection were less likely to test positive for SARS-CoV-2 upon admission for ARI compared to those who did not have evidence of prior infection (31/96 [32%] vs 673/1056 [64%]; adjusted OR: 0.25 [0.15, 0.41] (Table).

**Conclusion:**

Reinfections represented a small proportion (< 10%) of COVID-19-related hospitalizations. Prior SARS-CoV-2 infection provided meaningful protection against subsequent COVID-19-related hospitalization. The durability of this infection-induced immunity, variant-specific estimates, and the additive impact of vaccination are needed to further elucidate these findings.

**Disclosures:**

**Laura A. Puzniak, PhD. MPH**, Merck & Co., Inc.: Stocks/Bonds|Pfizer, Inc.: Stocks/Bonds **Robin Hubler, MS**, Pfizer Inc.: Employee|Pfizer Inc.: Stocks/Bonds **Srinivas Valluri, PhD**, Pfizer: Employee|Pfizer: Stocks/Bonds **Benjamin Lopman, PhD**, Epidemiological Research and Methods, LLC: Advisor/Consultant **Satoshi Kamidani, MD**, NIH: His institution (Emory University) receives funds from Pfizer for his work as a co-investigator on clinical trials of Pfizer COVID-19 vaccine.|Pfizer: His institution (Emory University) receives funds from Pfizer for his work as a co-investigator on clinical trials of Pfizer COVID-19 vaccine. **Christina A. Rostad, MD**, BioFire Inc, GSK, MedImmune, Micron, Merck, Novavax, PaxVax, Pfizer, Regeneron, Sanofi-Pasteur.: Grant/Research Support|Meissa Vaccines, Inc.: Co-inventor of RSV vaccine technology licensed to Meissa Vaccines, Inc.|NIH (Funding from NIH to conduct clinical trials of Moderna and Janssen COVID-19 vaccines): Grant/Research Support **John M. McLaughlin, PhD**, Pfizer: Employee|Pfizer: Stocks/Bonds **Evan J. Anderson, MD**, GSK: Advisor/Consultant|GSK: Grant/Research Support|Janssen: Advisor/Consultant|Janssen: Grant/Research Support|Kentucky Bioprocessing, Inc: Data Safety Monitoring Board|MedImmune: Grant/Research Support|Medscape: Advisor/Consultant|Merck: Grant/Research Support|Micron: Grant/Research Support|NIH: Funding from NIH to conduct clinical trials of Moderna and Janssen COVID-19 vaccines|PaxVax: Grant/Research Support|Pfizer: Advisor/Consultant|Pfizer: Grant/Research Support|Regeneron: Grant/Research Support|Sanofi Pasteur: Advisor/Consultant|Sanofi Pasteur: Grant/Research Support|Sanofi Pasteur: Data Adjudication and Data Safety Monitoring Boards|WCG and ACI Clinical: Data Adjudication Board.

